# A coordinated non-orthogonal multiple access strategy for integrated terrestrial-satellite networks

**DOI:** 10.1371/journal.pone.0248173

**Published:** 2021-03-30

**Authors:** Qiang He, Zheng Xiang, Peng Ren

**Affiliations:** Xidian University, Xi’an, China; IIT Madras, INDIA

## Abstract

In this paper, we investigate the outage probability and ergodic sum capacity of the downlink of the integrated satellite-terrestrial networks (ISTN) with a cooperative non-orthogonal multiple access (CNOMA) scheme, in which a user with better channel condition acts as a relay node and forwards information to the other users. In this paper, a pilot-based channel estimation method is considered which can verify the performance of this scheme with the imperfect channel state information. In this model, all these users are equipped with multi-antennas, and all of them are both in the coverage of a same beam of the satellite. Specifically, the exact analytical expression for the outage probability and ergodic sum capacity of the system is derived. The result shows that this coordinated non-orthogonal multiple access (CNOMA) scheme performs better than that of OMA (TDMA) in this model. Finally, the future research directions are given to further enhance the system capacity.

## 1. Introduction

In recent years, the satellite network is becoming more and more important for worldwide communication because of its global coverage, broadcasting capability and high bandwidth levels. The terrestrial network will be easily interconnected with other parts of the world by the satellite network. With the development of satellite network and terrestrial network, building an Integrated Terrestrial-Satellite Network (ITSN) to provide global internet access, which can realize communication whenever, and wherever, has been ever more attractive [1]. The integrated network consists of many heterogeneous space nodes including GEO, MEO, and LEO satellites and terrestrial network and gateways. The integrated network is a unified network similar to the terrestrial network, in which all nodes are interconnected by satellite-satellite links, satellite-gateway links, and gateway-users links, simultaneously.

Until now, the orthogonal multiple access (OMA) scheme is the main access scheme of ITSN [2]. For example, FDMA, TDMA, and CDMA are three common multiple access schemes which can provide services by fixed channel allocation. Similarly, the channel can be assigned randomly by ALOHA or be assigned according to the demand by demand assignment multiple access (DAMA) scheme, respectively. Although the application of orthogonal multiple access scheme can improve the performance of multiple access, such as it can avoid intra-beam interference and simplify the signal detection, however, for a single resource, it can not serve multiple users, which limits the further improvement of spectrum efficiency and capacity of satellite networks. However, a novel multiple access scheme, which is called power domain non-orthogonal multiple access (NOMA), has been proposed and it is generally considered to be the most potential multiple access scheme [3]. There are two key ideas of this new strategy, which are superposing multiple signals in the power domain at the transmitter and using of successive interference cancellation (SIC) at the receiver. Thus, the NOMA scheme can serve multiple users simultaneously on the same time/frequency block and provides an improved spectral efficiency at the cost of reasonable increased complexity [4].

With the development of integrated terrestrial-satellite networks, there is an urgent demand for massive machine-type terminals to get service by this networks. In recent decades, many scholars have begun to study the introduction of non-orthogonal multiple access into this integrated networks [5]. proposed a new scheme which is called non-orthogonal slotted aloha. This new scheme is used in the network which is TDMA-based random multiple access in internet of things satellite networks. This work utilized a new frame structure which is called intra-tile repetitions, and it can get a better performance [6]. proposed a new framework of application of non-orthogonal multiple access, which can apply it in the coordinated direct and relay transmission. This paper introduced a new scene where a base station directly communicates with user 1 (U1) while communicating with user 2 (U2) only through a relay, and then use the non-orthogonal multiple access in this scene. Analytical formulas of outage probability and ergodic sum capacity are given and the result reveals that it can get better performance with this new strategy in this network.

This work introduces a coordinated NOMA in ITN where NOMA plays a key role for acquiring the side information, when a satellite directly communicates with users in its coverage. The users are with different channel gain, and the user with better channel condition acts as a relay node and forwards information to the other user. The main contribution of this work can be described as follows.

1. Introduce the coordinated non-orthogonal multiple access strategy into a new scenario. According to the new scenario, a network is considered where U1 and U2 are in the same spot beam of a satellite, and U1 communicates with the satellite directly while U2 needs a help of U1. In this paper, U1 and U2 are all equipped with multi-antennas, which can improve the anti-jamming performance.
2. Analytical expressions for outage probability of each stream for U1 and U2 are derived, and then analytical expression for ergodic sum capacity is provided, too. These two parameters can well reflect the performance of CNOMA in this new scenario. Finally, the simulation results of these two parameters are given respectively to verify the performance of this new strategy.
3. Analysis of the effect of imperfect channel state information. In this paper, a pilot-based channel estimation method is considered which can verify the performance of this scheme with the imperfect channel state information. Finally, the simulation results are given to verify the impact of the imperfect channel state information.

## 2. System model

**Fig 1:**
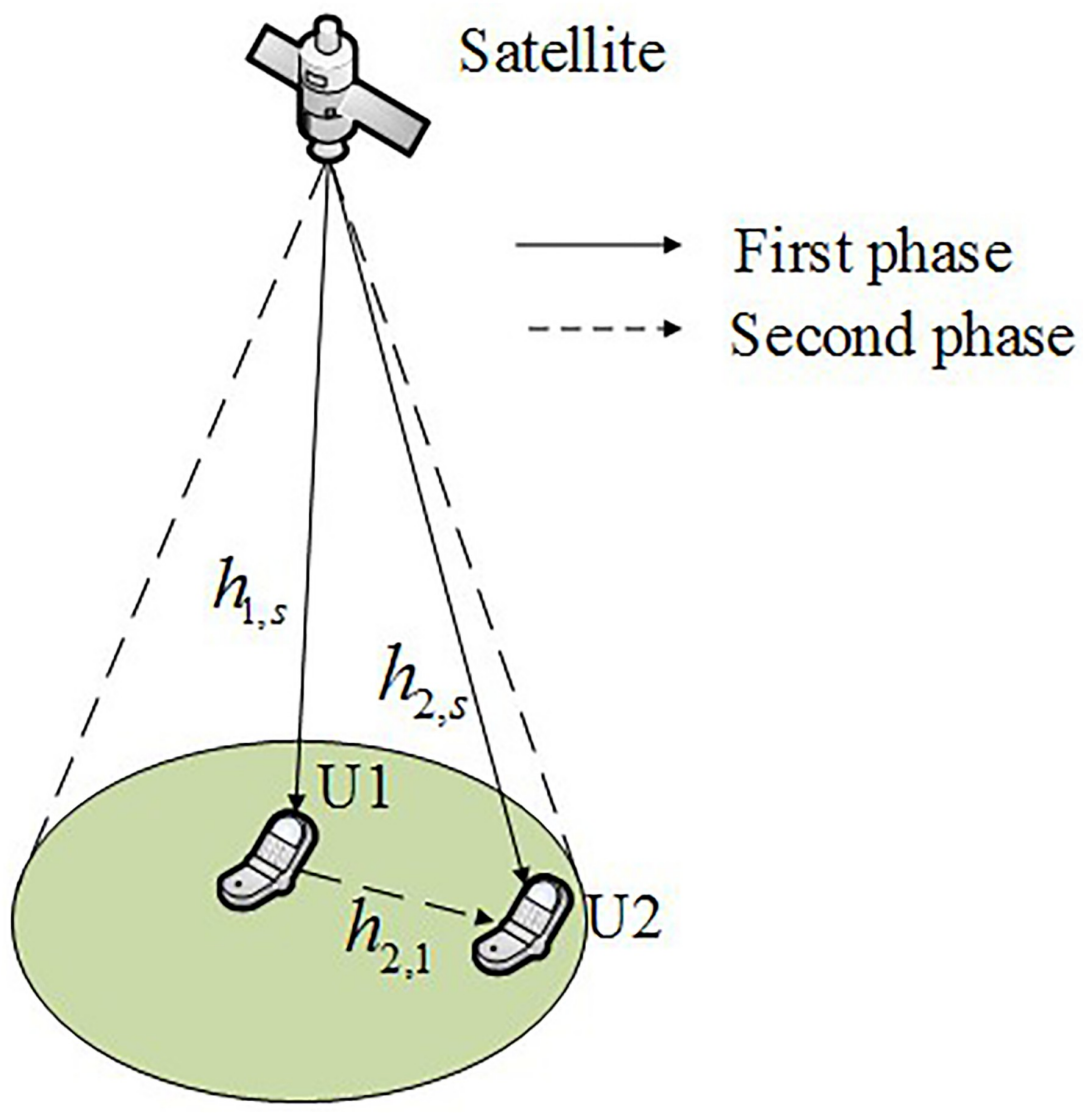
System model.

Fig 1 shows the system model. As illustrated in this figure, consider a system model with two pre-paired terrestrial users U1 and U2, where they are located in the same spot beam but with different locations of a low earth orbit satellite. Because of the location of U2, it can not receive the signal from satellite effectively, which need the forwarding of U1. We assume that U1 is with a better channel condition than that of U2. Hereafter, subscripts *s*, 1, and 2 denote the satellite, U1, and U2, respectively. U1 and U2 are both equipped with multi-antennas (number of antennas are *N*_1_ and *N*_2_, respectively), while *s* is equipped with a single antenna.$h_{2,s}\sim N(0,\sigma_{2,s}^{2})$, $h_{1,s}\sim N(0,\sigma_{1,s}^{2})$ and $h_{2,1}\sim N(0,\sigma_{2,1}^{2})$ are the channel coefficients from satellite to U2, satellite to U1, and U1 to U2, respectively. *h*_1,*s*_, *h*_2,*s*_ and *h*_2,1_ are *N*_1_ × 1, *N*_2_ × 1 and *N*_1_ × *N*_2_ channel vector, respectively. In this paper we divide the transmission into two phases. During the first phase, the satellite transmits the superposing signal to U1 and U2 directly, and during the second phase, U1 forwards the signal to U2.

During the first phase, we assume that *P*_*s*_ is the transmission power of the satellite. *λ*_1_(*t*_1_) and *λ*_2_(*t*_1_) are the power allocation coefficients for U1 and U2, where *λ*_1_(*t*_1_) + *λ*_2_(*t*_1_) = 1 and *λ*_1_(*t*_1_) < *λ*_2_(*t*_1_). It is assumed that the satellite transmits $x(t_{1})=\sqrt{P_{s}\lambda_{1}(t_{1})}x_{1}(t_{1})+\sqrt{P_{s}\lambda_{2}(t_{1})}x_{2}(t_{1})$, so the received signals at U1 and U2 can be denoted by (1). Where *G*_1,*s*_, *G*_2,*s*_,$n_{1}(t_{1})\sim N(0,\delta_{1,s}^{2})$ and $n_{2}(t_{1})\sim N(0,\delta_{2,s}^{2})$ are the gain of satellite to U1, the gain of satellite to U2, and the Additive White Gaussian Noise (AWGN) of the satellite to U1 and U2, respectively. One factor, in particular, needs highlighting: *G*_1,*s*_ and *G*_2,*s*_ is the parameter that contains the space loss which is caused by the imperfect channel from the transmitter to the receiver.$w^{H_{1}}$ and $w^{H_{2}}$ are the receive beamforming (BF) weight vector of the satellite to U1 and U2, respectively.

$$\begin{array}{l} S_{1}(t_{1})=\sqrt{P_{s}G_{1,s}}w^{H_{1}}h_{1,s}x(t_{1})+w^{H_{1}}n_{1}(t_{1})\\ S_{2}(t_{1})=\sqrt{P_{s}G_{2,s}}w^{H_{2}}h_{2,s}x(t_{1})+w^{H_{2}}n_{2}(t_{1})\\ w^{H_{1}}=\frac{h_{1,s}}{{\left\|h_{1,s}\right\|}^{F}},w^{H_{2}}=\frac{h_{2,s}}{{\left\|h_{2,s}\right\|}^{F}} \end{array}$$ (1)

In this paper, the down link of satellite to users is modeled as shadowed-Rician fading distribution [7]. The probability density function (PDF) of |*h*_1,*s*_|^2^ and |*h*_2,*s*_|^2^ are denoted by formula (2).

$$\begin{array}{l} f_{{\left|h_{1,s}\right|}^{2}}(x)=\lambda_{1,s}e^{-\omega_{1,s}x}F_{1}(m_{1,s};1;-\delta_{1,s}x)\\ f_{{\left|h_{2,s}\right|}^{2}}(x)=\lambda_{2,s}e^{-\omega_{2,s}x}F_{1}(m_{2,s};1;-\delta_{2,s}x)\\ \lambda_{1,s}=0.5{\left(2b_{1,s}m_{1,s}/\left(2b_{1,s}m_{1,s}+\Omega_{1,s}\right)\right)}^{m_{1,s}}/b_{1,s}\\ \lambda_{2,s}=0.5{\left(2b_{2,s}m_{2,s}/\left(2b_{2,s}m_{2,s}+\Omega_{2,s}\right)\right)}^{m_{2,s}}/b_{2,s}\\ \omega_{1,s}=1/2b_{1,s},\omega_{2,s}=1/2b_{2,s}\\ \delta_{1,s}=0.5\Omega_{1,s}/b_{1,s}/(2b_{1,s}m_{1,s}+\Omega_{1,s})\\ \delta_{2,s}=0.5\Omega_{2,s}/b_{2,s}/(2b_{2,s}m_{2,s}+\Omega_{2,s}) \end{array}$$ (2)

Where 2*b*_1,*s*_, 2*b*_2,*s*_, Ω_1,*s*_ and Ω_2,*s*_ are the average power of the multipath component and line-of-sight (LoS) component, respectively. *m*_1,*s*_ and *m*_2,*s*_ denote the Nakagami-m parameter of LoS. *F*_1_(.) is the confluent hypergeometric function.

According to NOMA, the user with low effective channel gain decodes its signal directly, and the user with better effective channel gain decodes its signal by SIC. Thus, the end-to-end SINR of U1 (*γ*_1_(*t*_1_)) and U2 (*γ*_2_(*t*_1_)) can be written as:

$$\begin{array}{l} \gamma_{1}(t_{1})=\frac{\lambda_{1}(t_{1})G_{1,s}P_{s}{\left|h_{1,s}\right|}^{2}}{\lambda_{2}(t_{1})G_{1,s}P_{s}{\left|h_{1,s}\right|}^{2}+\delta_{1,s}^{2}}\\ \gamma_{2}(t_{1})=\frac{\lambda_{2}(t_{1})G_{2,s}P_{s}{\left|h_{2,s}\right|}^{2}}{\lambda_{1}(t_{1})G_{2,s}P_{s}{\left|h_{2,s}\right|}^{2}+\delta_{2,s}^{2}} \end{array}$$ (3)

During the second time phase, U1 forwards the decoded information to U2. Meanwhile, the satellite transmits a new signal with power of *λ*_1_(*t*_2_)*P*_*s*_ and *λ*_2_(*t*_2_)*P*_*s*_ to U1 and U2, respectively. Then the received signals at U1 and U2 are:

$$\begin{array}{l} S_{1}(t_{2})=\sqrt{P_{s}\lambda_{1}(t_{2})G_{1,s}}w^{H_{1}}h_{1,s}x_{1}(t_{2})+w^{H_{1}}n_{1}(t_{2})\\ S_{2}(t_{2})=\sqrt{P_{s}\lambda_{2}(t_{2})G_{2,s}}w^{H_{2}}h_{2,s}x_{2}(t_{2})+\sqrt{P_{r}G_{r}}w^{H_{21}}h_{2,1}x_{2}(t_{1})+w^{H_{2}}n_{2}(t_{2})\\ w^{H_{1}}=\frac{h_{1,s}}{{\left\|h_{1,s}\right\|}^{F}},w^{H_{2}}=\frac{h_{2,s}}{{\left\|h_{2,s}\right\|}^{F}},w^{H_{21}}=\frac{h_{2,1}}{{\left\|h_{2,1}\right\|}^{F}} \end{array}$$ (4)

With the scheme of SIC, U1 and U2 can decoded the signals belong to them respectively.

Thus, the end-to-end SINR of U1 (*γ*_1_(*t*_2_)), U2 (*γ*_2_(*t*_2_)) and decoding SINR of U2 (*γ*_21_(*t*_2_)) can be written as:

$$\begin{array}{l} \gamma_{1}(t_{2})=\frac{\lambda_{1}(t_{2})P_{s}G_{1,s}{\left|h_{1,s}\right|}^{2}}{\lambda_{2}(t_{2})G_{2,s}P_{s}{\left|h_{1,s}\right|}^{2}+\delta_{2,s}^{2}}\\ \gamma_{21}(t_{2})=\frac{\lambda_{1}(t_{2})P_{s}G_{1,s}{\left|h_{1,s}\right|}^{2}}{\lambda_{1}(t_{2})G_{1,s}P_{s}{\left|h_{2,s}\right|}^{2}+\lambda_{2}(t_{2})G_{2,s}P_{s}{\left|h_{2,s}\right|}^{2}+\delta_{2,s}^{2}}\\ \gamma_{2}(t_{2})=\frac{P_{r}G_{r}{\left|h_{2,1}\right|}^{2}}{\lambda_{1}(t_{2})G_{1,s}P_{s}{\left|h_{2,s}\right|}^{2}+\lambda_{2}(t_{2})G_{2,s}P_{s}{\left|h_{2,s}\right|}^{2}+\delta_{2,s}^{2}} \end{array}$$ (5)

where *P*_*r*_ is the transmit power of U1, and *G*_*r*_ is the gain of U1 to U2.

The terrestrial links are modeled as independent and identically distributed Nakagami-m fading distributions. So, the PDF of |*h*_2,1_|^2^ can be given as:

$$\begin{array}{l} f_{{\left|h_{2,1}\right|}^{2}}(x)=\frac{m_{2,1}^{m_{2,1}N_{2,1}}x^{m_{2,1}N_{2,1}-1}}{\Gamma (m_{2,1}N_{2,1})\Omega_{2,1}^{m_{2,1}N_{2,1}}}e^{-\frac{m_{2,1}x}{\Omega_{2,1}}}\\ N_{2,1}=N_{1}\times N_{2} \end{array}$$ (6)

where Γ(.) is the Gamma function, *m*_2,1_ is the fading severity parameter, and Ω_2,1_ is the average power of U1 to U2 link.

## 3. Performance analysis

In this section, we investigate the outage performance and ergodic sum capacity of the coordinated NOMA strategy for ITSN. According to [8,9], the authors believe that it is important to study the outage probability when data rates of each stream are adjusted according to the quality of service (QoS) requirements of each user. But unlike the outage probability, the ergodic sum capacity is important when the transmission rates are predetermined in accordance with users’ channel conditions.

### 3.1 Outage probability

The outage probability is defined as the probability that the instantaneous SINR falls below a predefined threshold *γ*_*th*_, that is:

$$P_{out}(\gamma_{th})=\text{Pr}(\gamma \leq \gamma_{th})=F_{\gamma }(\gamma_{th})$$ (7)

where F_*γ*_(*γ*_*th*_) denotes the cumulative distribution function (CDF) of *γ*.

For U1, the outage event takes place only when either the received SINR of *γ*_1_(*t*_1_) falls below a threshold *γ*_*th*1_, or the decoding SINR *γ*_1_(*t*_2_) falls below a threshold *γ*_*th*1_, that is:

$$P_{out,1}^{}=1-\text{Pr}(\gamma_{1}(t_{1})\geq \gamma_{th1},\gamma_{1}(t_{2})\geq \gamma_{th1})$$ (8)

we can get (9) and (10),

$$P_{out,1}^{}=\{\begin{array}{l} \sum_{k=0}^{\infty }H_{1,s}\mu (k+1,\frac{\gamma_{th1}\omega_{1,s}\delta_{{}_{1,s}}^{2}}{P_{s}(1-\lambda_{1}(t_{1})-\lambda_{1}(t_{2})\gamma_{th1})})+\sum_{k=0}^{\infty }H_{1,s}\mu (k+1,\frac{\gamma_{th1}\omega_{1,s}\delta_{{}_{1,s}}^{2}}{\lambda_{1}(t_{2})P_{s}})[1-\sum_{k=0}^{\infty }H_{1,s}\mu (k+1,\frac{\gamma_{th1}\omega_{1,s}\delta_{{}_{1,s}}^{2}}{P_{s}(1-\lambda_{1}(t_{1})-\lambda_{1}(t_{2})\gamma_{th1})})],\\ \text{for}\frac{1-\lambda_{1}(t_{1})}{\lambda_{1}(t_{2})}>\gamma_{th1}\\ 1,\text{for}\frac{1-\lambda_{1}(t_{1})}{\lambda_{1}(t_{2})}\leq \gamma_{th1} \end{array}$$ (9)

$$\begin{array}{l} H_{1,s}=\frac{\alpha_{1,s}{\left(m_{1,s}\right)}_{k}\delta_{1,s}^{k}}{{\left(k!\right)}^{2}\omega_{1,s}^{k+1}}\\ {\left(a\right)}_{k}=\frac{\Gamma (a+k)}{\Gamma (a)}\\ u(x,y)=\int_{0}^{y}e^{-t}t^{x-1}dt \end{array}$$ (10)

where *u*(*x*, *y*) is the incomplete Gamma function. Then, we can substitute (9) into (8), and we can get the outage probability of U1.

For U2, the outage probability can be expressed as:

$$\begin{array}{l} P_{out,2}=1-[1-\text{Pr}(\gamma_{2}(t_{1})+\gamma_{2}(t_{2})\leq \gamma_{th2})][1-Pr(\gamma_{21}(t_{2})\leq \gamma_{th1})]\\ =1-[1-F_{\gamma_{2}(t_{1})+\gamma_{2}(t_{2})}\text{(}\gamma_{th2}\text{)][1-}F_{\gamma_{21}(t_{2})}\text{(}\gamma_{th1}\text{)]} \end{array}$$ (11)

$$F_{\gamma_{2}(t_{1})+\gamma_{2}(t_{2})}\text{(}\gamma_{th2}\text{)}=\text{Pr}(\gamma_{2}(t_{1})\leq \gamma_{th2}-\gamma_{2}(t_{2})\leq \gamma_{th2})$$ (12)

$$F_{\gamma_{2}(t_{1})+\gamma_{2}(t_{2})}=\{\begin{array}{l} \int_{0}^{\Delta }F_{{\left|h_{2,s}\right|}^{2}}[g(z)]f_{{\left|h_{2,1}\right|}^{2}}(z)dz,for\frac{1-\lambda_{2}(t_{1})}{\lambda_{2}(t_{2})}\geq \gamma_{th2}\\ \int_{0}^{\Delta (1-\frac{1-\lambda_{2}(t_{2})}{\lambda_{2}(t_{2})\gamma_{th2}})}f_{{\left|h_{2,1}\right|}^{2}}zdz+\\ \int_{\Delta }^{\Delta }(1-\frac{1-\lambda_{2}(t_{1})}{\lambda_{2}(t_{2})\gamma_{th2}})F_{{\left|h_{2,s}\right|}^{2}}g(z)f_{{\left|h_{2,1}\right|}^{2}}zdz,\text{for}\frac{1-\lambda_{2}(t_{1})}{\lambda_{2}(t_{2})}<\gamma_{th2} \end{array}$$ (13)

$$\begin{array}{l} g(z)=\frac{\gamma_{th2}\delta_{2,s}^{2}(1-z/\Delta )}{P_{s}(1-\lambda_{2}(t_{2})\gamma_{th2}(1-z/\Delta ))}\\ \Delta =\frac{\gamma_{th2}\delta_{2,1}^{2}}{P_{r}}\\ F_{{\left|h_{2,s}\right|}^{2}}[g(z)]=\sum_{k=0}^{\infty }H_{2,s}\mu (k+1,g(z)\omega_{2,s}) \end{array}$$ (14)

we can substitute (12)–(14) into (11), and then we can get the outage probability of U2.

With the CDFs of *h*_1,s_, *h*_2,s_, *h*_2,1_, we can rewrite the *P*_*out*,1_ and *P*_*out*,2_ by a unified form as:

$$\begin{array}{l} P_{out,k}^{}=F_{{\left|h_{k,s}\right|}^{2}}(\eta_{k}^{*})+F_{{\left|h_{2,1}\right|}^{2}}(\tau_{21}^{*})-F_{{\left|h_{ks}\right|}^{2}}(\eta_{k}^{*})+F_{{\left|h_{2,1}\right|}^{2}}(\tau_{21}^{*})\\ \eta_{1}^{*}=\frac{\gamma_{th1}(\frac{\lambda_{1}(t_{1})}{LS_{1}}+1)}{P_{s}G_{1,s}/\sigma_{1,s}^{2}(\lambda_{1}(t_{1})-\gamma_{th1}\lambda_{2}(t_{1}))}\\ \eta_{2}^{*}=\{\eta_{1}^{*},\frac{\gamma_{th2}(\frac{1}{LS_{1}}+1)}{P_{s}G_{2,s}\lambda_{2}(t_{1})}\}\\ \tau_{21}^{*}=\frac{\gamma_{th2}(\frac{1}{LS_{2}}+1)}{P_{r}\lambda_{2}(t_{2})} \end{array}$$ (15)

where *LS*_1_, *LS*_2_ are the length of the pilot of the link from the satellite to the users, and U1 to U2 respectively.

### 3.2 Ergodic sum capacity

In this section, we will discuss the ergodic sum capacity of the proposed strategy. Easily, we can draw that the data rate of users are denoted by (16):

$$\begin{array}{l} R_{1}(t_{1})=\frac{1}{2}{\text{log}}_{2}(1+\gamma_{1}(t_{1}))\\ R_{1}(t_{2})=\frac{1}{2}{\text{log}}_{2}(1+\gamma_{1}(t_{2}))\\ R_{2}(t_{1})=\frac{1}{2}{\text{log}}_{2}(1+\gamma_{2}(t_{1}))\\ R_{2}(t_{2})=\frac{1}{2}{\text{log}}_{2}(1+\gamma_{2}(t_{2}))\\ R_{21}(t_{2})=\frac{1}{2}{\text{log}}_{2}(1+\gamma_{21}(t_{2})) \end{array}$$ (16)

where *R*_1_(*t*_1_), *R*_1_(*t*_2_), *R*_2_(*t*_1_), *R*_2_(*t*_2_) and *R*_21_(*t*_2_) are the rate of satellite transmits to U1 during the first phase, the second phase, satellite transmits to U2 during the first phase, the second phase, and U1 transmits to U2 during the second phase, respectively.

In this section, we suppose that the threshold SINRs of all users’ are adjusted according to users’ channel conditions, the ergodic sum capacity of the proposed scheme is given by (17):

$$R_{esc}=\text{E}[R_{1}(t_{1})]+\text{E}[R_{1}(t_{2})]+\text{E}[R_{2}(t_{1})]+\text{E}[R_{2}(t_{2})]+\text{E}[R_{21}(t_{2})]$$ (17)

where E(*X*) means the expectation of a random variable of *X*. By the definition given by (18):

$$\begin{array}{l} \int_{0}^{\infty }\frac{1}{2}{\text{log}}_{2}(1+x)f_{X}(x)dx=\frac{1}{2\text{ln}2}\int_{0}^{\infty }\frac{1-F_{X}(x)}{1+x}dx\\ F_{\gamma_{1}(t_{1})}(x)=1-e^{-\frac{x}{\lambda_{1}(t_{1})P_{s}\sigma_{1,s}^{2}}}\\ F_{\gamma_{2}(t_{1})}(x)=1-e^{-\frac{x}{\lambda_{2}(t_{1})P_{s}\sigma_{2,s}^{2}}}\\ F_{\gamma_{21}(t_{2})}(x)=1-e^{-\frac{x}{P_{r}\sigma_{2,1}^{2}}}\\ F_{\gamma_{1}(t_{2})}(x)=1-e^{-\frac{x}{\lambda_{1}(t_{2})P_{s}\sigma_{1,s}^{2}}}\\ F_{\gamma_{2}(t_{2})}(x)=1-e^{-\frac{x}{\lambda_{2}(t_{2})P_{s}\sigma_{2,s}^{2}}} \end{array}$$ (18)

*E*(*R*_1_(*t*_1_)), *E*(*R*_2_(*t*_1_)), *E*(*R*_1_(*t*_2_)), *E*(*R*_2_(*t*_2_)), *E*(*R*_21_(*t*_2_)) can be denoted by (19):

$$\begin{array}{l} E(R_{1}(t_{1}))=-\frac{1}{2\text{ln}2}e^{\frac{1}{\lambda_{1}(t_{1})P_{s}\sigma_{1,s}^{2}}}I(-\frac{1}{\lambda_{1}(t_{1})P_{s}\sigma_{1,s}^{2}})\\ E(R_{2}(t_{1}))=-\frac{1}{2\text{ln}2}e^{\frac{1}{\lambda_{2}(t_{1})P_{s}\sigma_{2,s}^{2}}}I(-\frac{1}{\lambda_{2}(t_{1})P_{s}\sigma_{2,s}^{2}})\\ E(R_{21}(t_{2}))=-\frac{1}{2\text{ln}2}e^{\frac{1}{P_{r}\sigma_{2,1}^{2}}}I(-\frac{1}{P_{r}\sigma_{2,1}^{2}})\\ E(R_{1}(t_{2}))=-\frac{1}{2\text{ln}2}e^{\frac{1}{\lambda_{1}(t_{2})P_{s}\sigma_{1,s}^{2}}}I(-\frac{1}{\lambda_{1}(t_{2})P_{s}\sigma_{1,s}^{2}})\\ E(R_{2}(t_{2}))=-\frac{1}{2\text{ln}2}e^{\frac{1}{\lambda_{2}(t_{2})P_{s}\sigma_{2,s}^{2}}}I(-\frac{1}{\lambda_{2}(t_{2})P_{s}\sigma_{2,s}^{2}})\\ I(x)=\int_{-\infty }^{x}\frac{e^{t}}{t}dt,x<0 \end{array}$$ (19)

Thus, (15) can be obtained according to (17).

## 4. Numerical results

In this section, numerical results are provided to evaluate the performance of the coordinated NOMA for the integrated terrestrial-satellite network as well as the algorithm proposed in this paper. The carrier frequency is set as 2 GHz at the S band and *G*_1,s_ = *G*_2,s_ = 52*dBi*. *m* = *m*_1,s_ = *m*_2,s_ = 5, *b*_1,s_ = *b*_2,s_ = 0.126, Ω_1,s_ = Ω_2,s_ = 0.835 *γ*_*th*1_ = −3dB, and *γ*_*th*2_ = −3dB According to the NOMA scheme, the user with worse channel gain can be allocated with more power resource. Thus, in this paper we consider *λ*_2_(*t*_1_) = 0.95, *λ* = *λ*_1_(*t*_1_) = 0.05, *LS*_1_ = *LS*_2_ = *LS* = 5. The satellite is assumed to be on the orbit of 1100 km, and the parameters of the satellite is defined referring to [10]. The maximum transmit power of U1 is set as *P*_*r*,max_ = 40*dBm*. The ground channel is assumed to be independent and identically distributed Nakagami-m fading, and is modeled referring to [11], while the satellite channel is modeled referring to [12].

**Fig 2:**
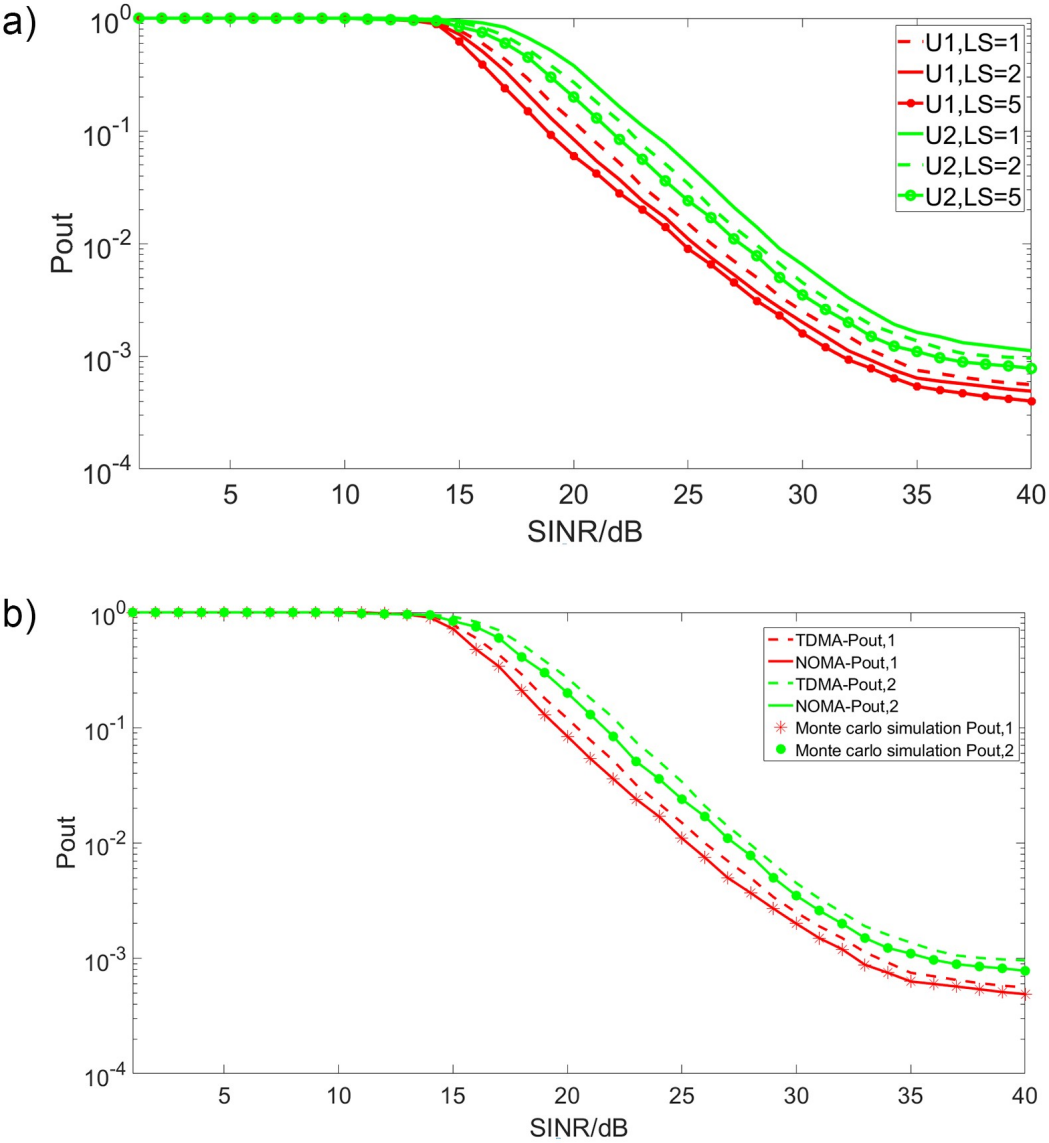
(a). Comparison of Outage Performance. (b). Impact of *LS*.

Fig 2 shows the outage performance of this new strategy proposed in this paper. Fig 2(a) is the outage performance which illustrates the comparison result of the outage performance between the analytical result and Monte carlo simulation result. This figure also gives the comparison of the outage performance with TDMA which is an OMA that always used in the satellite communication networks. According to this figure, we can draw that the analytical results agree well with the Monte Carlo simulations. The outage performance of user 1 is much better especially when SINR>15dB. With the increase of SINR, the outage performance of user 1 will be improved more significantly than that of user 2. Another obvious conclusion is that the performance of CNOMA is better than TDMA. Fig 2(b) is the impact of the length of the pilot which shows the effect of the imperfect channel state information. In this figure, the performance of the system becomes better with the increase of *LS* which is in the range of 1 and 5. Thus, we can draw that: 1) since the better channel condition, the outage performance of user 1 outperforms user 2, 2) the outage performance of CNOMA is better than OMA in this integrated satellite-terrestrial networks, 3) with the condition of imperfect channel state information, the performance will be much better when the length of polite increase in a specific range.

**Fig 3:**
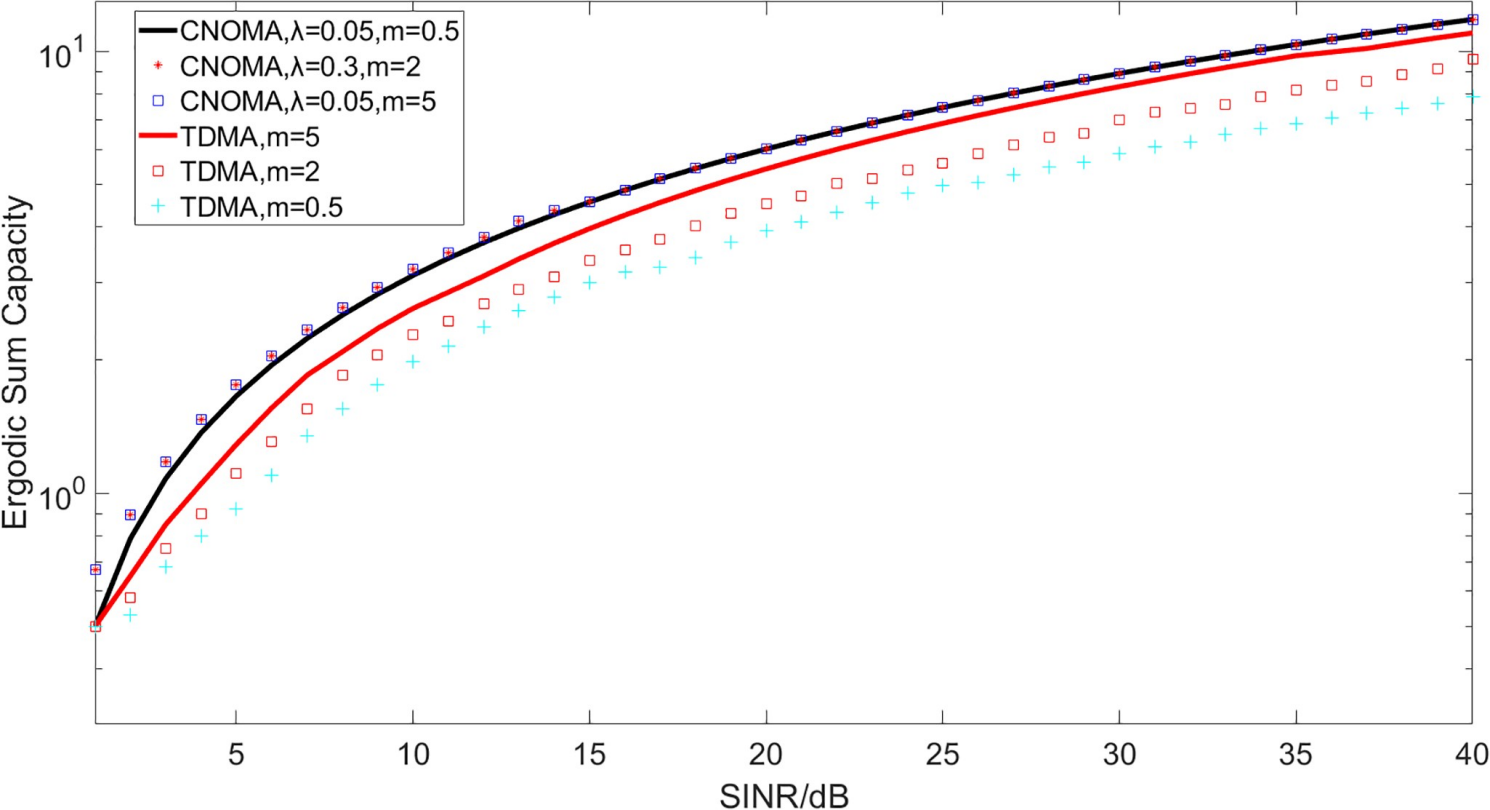
Ergodic sum capacity.

Fig 3 is the ergodic sum capacity which shows the ergodic sum capacity according to SINR with the same parameters used in Fig 2. In this figure, the ergodic sum capacity of the CNOMA scheme is compared to an OMA scheme (TDMA) in ISTN with different values of *λ* (the power allocation coefficients for U1, and the power allocation coefficients for U2 is 1-*λ*) and *m* (the Nakagami-m parameter of LoS). The results show that the CNOMA scheme provides outstanding performance over the TDMA scheme in ISTN by enabling the transmission of a user during the second phase. Meanwhile, it shows that the proposed NOMA scheme outperforms OMA which is because that the transmission slots needed by the TDMA scheme are twice of those needed by the CNOMA scheme. In this figure, we can see that it have little effect on the proposed system performance with the change of *λ* and m. It can be observed that: 1) the performance of the ergodic sum capacity for the proposed scheme is much better than that of OMA, 2) the ergodic sum capacity can increase along with the SNR, 3) the power allocation coefficients for users and the Nakagami-m parameter of LoS have little effect on the ergodic sum capacity in this system.

## 5. Conclusions

In this paper, we have investigated a general downlink framework of the coordinated non-orthogonal multiple access (CNOMA) based integrated terrestrial-satellite network, in which the satellite provide service for ground users. A pilot-based channel estimation method is considered here which can verify the performance of this scheme with the imperfect channel state information. The basic idea of this new scheme is to use non-orthogonal transmission at the transmitter, and realize correct demodulation by serial interference cancellation (SIC) at the receiver. We have mainly studied the downlink outage probability and ergodic sum capacity of this network. The result shows that the coordinated non-orthogonal multiple access (CNOMA) based network performs better than the OMA based network. For this reason, we suppose that we can introduce CNOMA into ISTN to improve network performance. AS a future work, we are considering a novel scene that with multiple small cells and macro cells on the ground to verification the performance of the new scheme.

## References

[1] Li Hewu et al. Progress and tendency of space and earth integrated network. Science and Technology Review. 2016, vol. 34, no. 14, pp. 95–106.

[2] Lutz E, Werner M, Jahn A. Satellite Systems for Personal and Broadband Communications. Springer-Verlag: Berlin, Germany, 2000.

[3] Zhu X., Jiang C., Kuang L., Ge N. and Lu J. Non-Orthogonal Multiple Access Based Integrated Terrestrial-Satellite Networks. IEEE Journal on Selected Areas in Communications.2017, vol. 35, no. 10, pp. 2253–2267.

[4] Saito Y, Kishiyama Y, Benjebbou A, Nakamura T, Li A, Higuchi K. Non-orthogonal multiple access (NOMA) for cellular future radio access. IEEE VTC. 2013, pp. 1–5.

[5] Wang Qiwei et al. A Framework of Non-Orthogonal Slotted Aloha (NOSA) Protocol for TDMA-Based Random Multiple Access in IoT-Oriented Satellite Networks. IEEE ACCESS. 2018, vol. 6, pp. 77542–77553.

[6] Kim Jung-Bin, Lee In-Ho. Non-Orthogonal Multiple Access in Coordinated Direct and Relay Transmission. IEEE COMMUNICATIONS LETTERS. 2015, vol. 19, no. 11, pp. 2037–2040.

[7] Yue Xinwei, et al. Outage behaviors of NOMA-based satellite network over Shadowed-Rician fading channels. IEEE Transactions on Vehicular Technology. 2020, vol. 69, no. 6, pp. 6818–6821.

[8] Jung-Bin K, In-Ho L. Non-Orthogonal Multiple Access in Coordinated Direct and Relay Transmission. IEEE Communications Letters. 2015, vol. 19, no. 11, pp. 2037–2040.

[9] Xie Silin, et al. Outage performance of NOMA-based integrated satellite-terrestrial networks with imperfect CSI. Electronics Letters. 2019, vol.55, no.14, pp. 793–795.

[10] Christopoulos D, Chatzinotas S, Ottersten B. Multicast multigroup precoding and user scheduling for frame-based satellite communications. IEEE Trans. Wireless Commun. 2015, vol. 14, no. 9, pp. 4695–4707.

[11] Further Advancements for E-UTRA Physical Layer Aspects. 3rd Generation Partnership Project (3GPP). 2016.

[12] Lutz E, Cygan D, Dippold M, Dolainsky F, Papke W. The land mobile satellite communication channel-recording, statistics, and channel model. IEEE Trans. Veh. Technol. 2013, vol. 40, no. 2, pp. 375–386.

